# Role of Adropin in Cardiometabolic Disorders: From Pathophysiological Mechanisms to Therapeutic Target

**DOI:** 10.3390/biomedicines9101407

**Published:** 2021-10-07

**Authors:** Josko Bozic, Marko Kumric, Tina Ticinovic Kurir, Ivan Males, Josip A. Borovac, Dinko Martinovic, Marino Vilovic

**Affiliations:** 1 Department of Pathophysiology, University of Split School of Medicine, 21000 Split, Croatia; marko.kumric@mefst.hr (M.K.); tticinov@mefst.hr (T.T.K.); jborovac@mefst.hr (J.A.B.); dinko.martinovic@mefst.hr (D.M.); marino.vilovic@mefst.hr (M.V.); 2 Department of Endocrinology, Diabetes and Metabolic Diseases, University Hospital of Split, 21000 Split, Croatia; 3 Department of Surgery, University Hospital of Split, Spinčićeva 1, 21000 Split, Croatia; ivan.males@live.com; 4 Department of Cardiology, University Hospital of Split, Spinčićeva 1, 21000 Split, Croatia

**Keywords:** adropin, endothelial dysfunction, cardiovascular disease, pathophysiology, biomarker

## Abstract

Although a large amount of data supports the crucial role of endothelial dysfunction (ED) in cardiovascular diseases (CVDs), there is a large bench-to-bedside chasm between basic and clinical research of ED, limiting the implementation of these findings in everyday clinical settings. Hence, it is important to further investigate the pathophysiological mechanisms underlying ED and find modalities that will alleviate its clinical implementation. Adropin, a highly conserved peptide hormone secreted primarily by the liver, recently emerged as an important regulatory component of the vascular endothelium. Specifically, the vasoprotective role of adropin is achieved mainly by affecting endothelial NO synthesis. Thus, in this review, we aimed to summarize the current knowledge regarding the role of adropin in physiological processes and address the protective role of adropin in endothelium with consequent implications to CV pathologies. We focused on data regarding the role of adropin in the clinical setting, with concurrent implications to future clinical use of adropin. Studies suggest that plasma levels of adropin correlate with indices of ED in various pathologies and enhanced disease progression, implying that adropin may serve as a useful biomarker of ED in the upcoming future. On the other hand, despite notable results with respect to therapeutic potential of adropin in preliminary experiments, further well-designed studies are warranted in order to establish if adropin might be beneficial in this setting.

## 1. Introduction

Evolving evidence suggests that there are virtually no cardiovascular diseases (CVDs) in which endothelial dysfunction (ED) is not implicated, at least in part [1]. In fact, to address the importance of endothelium in CVDs, endothelium has even been considered an organ system by multiple authors [2]. However, despite an abundant amount of data, there is a large bench-to-bedside chasm between basic and clinical research of ED, which probably reflects the inflexible and largely outdated nature of the present-day medical infrastructure. Moreover, multiple pathways underlying the association between ED and diverse CV, inflammatory, metabolic and infectious diseases have been established, yet the relative weight of each respective component still remains to be elucidated, as well as whether some of these could have clinical implications. Thus, it is important to further investigate the pathophysiological mechanisms underlying ED and, rather, more importantly, find modalities which will alleviate drawing inferences regarding ED in everyday clinical practice. Circulating biomolecules could be a promising niche for the latter, as they are relatively cheap and simple to use. Nevertheless, interpretation of laboratory findings is often challenging, as it is hard to find biomarkers of appropriate characteristics, as we discussed further in the text.

Adropin, a highly conserved 76 amino peptide hormone, secreted primarily by the liver, recently emerged as an important regulatory component of the vascular endothelium [3]. Apart from the well-established role of adropin in metabolic regulation, particularly with regard to glucose metabolism and insulin sensitivity in the heart, liver and skeletal muscle, accumulating data suggest that adropin has important implications on CV pathology [4,5,6]. In fact, stimulation of adropin-mediated pathways has been shown to improve endothelial function and reduce inflammation, thus ameliorating its detrimental effects. Notwithstanding, we are still far from implementing adropin in routine clinical practice, especially in terms of therapy, as therapeutic delivery of peptide hormones is restricted by their pharmacokinetic properties [7].

In this review, we aimed to summarize the current knowledge regarding the role of adropin in physiological process, but in a myriad of pathological processes as well. In particular, we addressed the protective role of adropin in the endothelium, with consequent therapeutic implications for CV pathologies.

## 2. Adropin

Adropin is a 76 amino acid peptide, secreted primarily by the liver and brain [8]. Amino acids from 1 to 33 are considered to encode the secretory signal peptide, whereas residues from 34 to 76 represent the biologically active secreted domain. Importantly, adropin is considered preserved, as the 76-amino acid long sequence in human, mouse and rat is 100% identical [9]. This hepatokine is encoded by the Energy Homeostasis-Associated (*Encho*) gene, which is mainly expressed in the two above-noted organs, especially in areas of the brain involved in metabolic regulation [10]. Adropin was first identified by Kumar et al. by using microarray screening of genes in melanocortin-3 receptor-deficient (*Mc3r^−/−^*) mice with hypothalamic obesity [9]. By using the transgenic overexpression of the open reading frame (ORF) and a synthetic peptide mimicking the effects of adropin (adropin^(34–76)^), the authors showed significant improvement in glucose homeostasis, lipid metabolism and weight loss in mice. Thus, the authors concluded that aside from its role in energy homeostasis, adropin may be involved in the metabolic adaptation to fasting and dietary macronutrients. However, it remains to be determined if the observed is a consequence of paracrine effects on hepatocytes or endocrine signals of macronutrient consumption and energy status.

**Figure 1:**
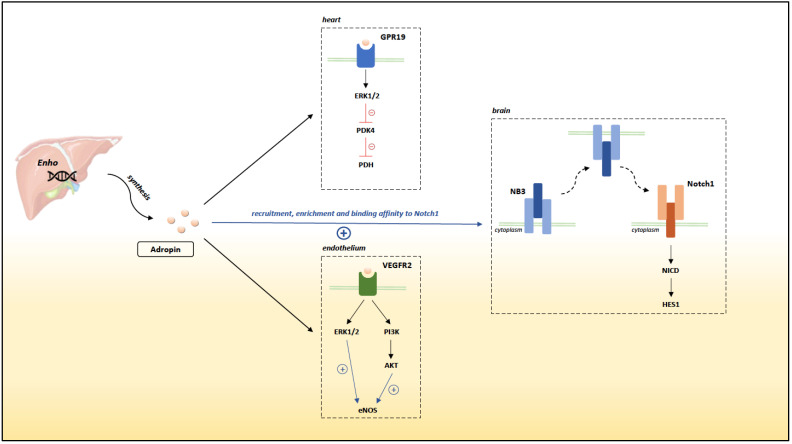
Receptors for adropin in different tissues. The blue “+” indicates stimulation, whereas red “−“ indicates inhibition. Abbreviations: GPR19: G-protein coupled receptor 19; VEGFR2: Vascular endothelial growth factor receptor 2; ERK 1/2: extracellular signal-regulated kinase 1/2; PI3K: Phosphoinositide 3-kinase; PDK4: pyruvate dehydrogenase kinase 4; PDH: pyruvate dehydrogenase; AKT: protein kinase B; NICD: intracellular domain of Notch; HES1: hairy and enhancer of split-1.

Further studies found that adropin acts as a secretory peptide, exerting its effects through extracellular receptors. So far, three distinct membrane receptors have been reported to bind adropin, depending on the target tissue (Figure 1) [7]. Wong et al. demonstrated that adropin acts via the Nb-3/Notch signaling pathway in mice brains [11]. Namely, adropin promotes recruitment, enrichment and binding affinity of Nb-3 to Notch1. Interestingly, the authors demonstrated that both adropin KO mice and Nb-3 KO mice have displayed similar impairment in motor coordination and synapse formation in the cerebellum, thus suggesting that adropin regulates physical activity and motor coordination through the aforementioned Nb-3/Notch signaling pathway. In addition, Gao et al. demonstrated that adropin induces expression of HES1, a target of Notch1 signaling, implying a link between adropin function and the above-noted pathway beyond the brain [12]. On the other hand, recent data suggest that adropin exhibits a central inhibitory effect on water deprivation-induced drinking [13]. This effect seems to be mediated by an orphan G-protein coupled receptor, GPR19. In fact, using small interfering RNA-mediated GPR19 gene silencing, Stein et al. demonstrated in vivo that the effect of adropin on central physiological regulation of water drinking is GPR19-dependent [13]. Furthermore, Rao et al. investigated the pathophysiological role of GPR19 in metastatic breast cancer and found that adropin activates GPR19 via the mitogen-activated protein kinase/extracellular signal-regulated kinase 1/2 (MAPK/ERK1/2) pathway, resulting in phenotypic and functional changes, mainly with respect to changes in cell invasion [14]. This aforementioned pathway was explored in a recent study by Thapa et al. [15]. The authors demonstrated that adropin regulates pyruvate dehydrogenase (PDH) in cardiac-derived H9c2 cells via a newly established GPR19-MAPK-PDK4 pathway. Namely, activation of GPR19 with adropin leads to MAPK-mediated phosphorylation that results in downregulation of pyruvate dehydrogenase kinase 4 (PDK4) and consequent disinhibition of PDH, an important regulator of cardiac cell bioenergetics. Knockout of GPR19 in mice has abrogated the adropin-induced effect on PDH, thus suggesting that GPR19 is a putative receptor for adropin in cardiac cells. Finally, favorable effects of adropin on endothelial function, which are further discussed, seem to be mediated mainly through Vascular endothelial growth factor receptor 2 (VEGFR2) [16].

## 3. Role of Adropin in Metabolism and Diverse Pathologies

The above-noted regulation of PDH activity seems to be the most important physiological role of adropin. It was demonstrated on adropin-KO mice models that adropin mediates substrate oxidation preference in skeletal muscles [17]. Specifically, adropin-KO mice preferentially oxidized fat over carbohydrates in comparison to adropin transgenic mice, by changing the expression of CD36 and carnitine palmitoyltransferase-1B (CPT1B) activity, key enzymes in fatty acid utilization, and PDH, a rate-limiting enzyme in glucose oxidation [18]. Additionally, Gao et al. demonstrated that adropin improves glucose tolerance and mitigates insulin resistance in a model of mice with diet-induced obesity, mediated by the aforementioned GPR19-MAPK-PDK4 pathway [17].

Accumulated data indicate that adropin contributes to the modulation of adiposity and metabolism of lipids and glucose in various tissues. Adropin-overexpressing mice challenged with a high-fat diet were protected from body weight gain in the first 6–8 weeks, but not after 3 months of high-fat diet, thus suggesting that adropin overexpression delays but does not completely prevent diet-induced body weight gain [9]. In addition, these mice had lower fasting levels of insulin and triglyceride but unchanged glucose levels in comparison to wild type mice, providing evidence that adropin overexpression improves insulin sensitivity and glucolipid metabolism in obesity. Multiple studies explored the direct effects of adropin on glucose and lipid metabolism. Thapa et al. demonstrated that basal and insulin-induced glucose production were low in the livers of diet-induced obesity mice treated with adropin (450 nmol/kg) for three days [19]. Furthermore, Gao et al. demonstrated that exogenously administered adropin increases hepatic insulin sensitivity and that suppressed liver glucose production is cAMP/Protein kinase A-mediated [20]. In skeletal muscles, low adropin is associated with increased fatty acid oxidation, whereas overexpression of adropin stimulates glucose oxidation and reduces lipid oxidation in a process mediated by PGC-1α [12]. Finally, in adipose tissue, adropin has been shown to suppress lipogenic genes, as demonstrated on a mouse model of obesity [9]. Furthermore, adropin stimulates proliferation of preadipocytes, but inhibits their differentiation into mature adipocytes [21].

As adropin is an important cog in the regulation of the glucose and lipid metabolism, the fact that adropin is implicated in the pathophysiology of various metabolic diseases should not be a surprise. Evidence suggests that adropin levels may be affected in diabetes mellitus (DM). Apart from being lower in patients with type 2 DM, lower adropin levels represent a risk factor for ED in these patients [4]. Similarly, lower levels of adropin in circulation were also found in women with gestational diabetes and children with type 1 DM [22,23]. On the contrary, several authors reported higher levels of adropin in diabetes, both in humans and in rats. Hosseini et al. reported higher serum levels of adropin in type 2 DM patients [24]. Furthermore, Aydin et al. explored the effects of type 1 DM induction on adropin levels in rats [25]. The authors demonstrated that rats with induced type 1 DM exhibit higher serum and tissue (pancreas, liver, kidney, brain, and cerebellum) levels of adropin than the control group. Overall, the data concerning the concentration of adropin in both types of diabetes are rather conflicting. Jasaszwili et al. argue that the observed discrepancy may be owing to species differences (rats vs. humans), progress of disease, and age of patients (children vs. adults) [4].

Diabetic cardiomyopathy (DCM), an under-recognized and lethal complication of diabetes, is commonly defined as cardiomyopathy in patients with diabetes mellitus (DM) in the absence of coronary artery disease, hypertension or dyslipidemia [26]. Among many others, diminished GLUT4 expression and increased CD36 expression, which lead to additional myocardial metabolism shift from glucose to free fatty acid (FFA) oxidation and consequent metabolic inflexibility, represent the pathophysiological mechanisms underlying this complex clinical entity [27]. As adropin reverses this phenotype in skeletal muscle of ex vivo hearts from lean non-fasting mice and diet-induced obesity mice, and as adropin levels are significantly lower in patients with DM, it is plausible that stimulation of adropin pathways could mitigate the detrimental effects of this lethal DM complication [3,17]. In line with this, our previous study showed that treating male obese type 2 DM patients with liraglutide, an incretin mimetic, results in a significantly higher plasma adropin concentration and improved indices of insulin resistance [28]. Although further studies are needed owing to lack of available data, we can hypothesize that the missing link could be endothelial dysfunction, as liraglutide has been shown to ameliorate endoplasmic reticulum stress and restore insulin-mediated endothelial nitric oxide synthase (eNOS) activation in endothelial cells [29]. In line with this, it is important to address that both GLP-1 agonists and adropin exhibit anti-inflammatory effects, thus including inflammation in the “equation” [4,30]. Finally, as adropin plasma levels in humans are influenced by obesity and dietary preferences (there is a positive association between human serum adropin levels and fat intake and a negative association with carbohydrate intake), liraglutide-induced changes in metabolic profile might be the explanation of this observation [31]. Of important note, it remains elusive whether diet affects adropin levels or adropin influences nutritional habits. Therefore, an association of adropin with diet preferences needs to be discussed with caution. Moreover, adropin treatment improved ex vivo cardiac function and efficiency, in an experiment by Altamimi et al. [3]. Namely, the authors demonstrated that ex vivo adropin infusion increases insulin signaling and sensitivity, thereby contributing to a more favorable metabolic profile of the heart.

Furthermore, plasma adropin concentrations significantly correlate with indices of disease severity in patients with obstructive sleep apnea (OSA), as we demonstrated in our previous study [32]. Moreover, in a study performed on pediatric OSA population, it has been shown that plasma adropin concentrations are reduced in pediatric patients with OSA in comparison to matched controls, especially when associated with ED [33]. Interestingly, adenotonsillectomy in children with both OSA and ED increased mean adropin levels but not in children with OSA without ED. The authors explained the presence of ED in some but not in all children with OSA (despite comparable severity) by recent exploration of epigenetic changes in the eNOS gene promoter, which suggest the presence of complex interactions between OSA and the vasculature. Nevertheless, it was concluded that adropin plasma levels in children with OSA may, in fact, provide a reliable indicator of the presence or absence of reduced NO bioavailability in the endothelium, i.e., a surrogate biomarker for the presence of ED in pediatric patients with OSA. Specifically, it was demonstrated that a cut-off value of adropin < 4.2 ng/mL provided 100% sensitivity and 94% specificity in detection of ED in children with OSA.

Furthermore, in a group of patients with inflammatory bowel disease (IBD), adropin levels were lower in comparison to the control group, and adropin also showed a significant negative correlation with multiple IBD severity scores [34]. The mechanistic explanation of these findings was twofold. The first included ED, one of the hallmarks of IBD and the other was the nuclear factor erythroid 2-related factor 2 (Nrf2)-mediated antioxidant effect of adropin.

In our previous study conducted on a population of patients undergoing hemodialysis, adropin levels were significantly lower in comparison to controls [35]. Moreover, there was a significant negative correlation between adropin and malnutrition inflammation score, dialysis malnutrition score, duration of hemodialysis and C-reactive protein, altogether implying the involvement of adropin in one of the many pathophysiological mechanisms underlying chronic kidney disease [36].

**Figure 2:**
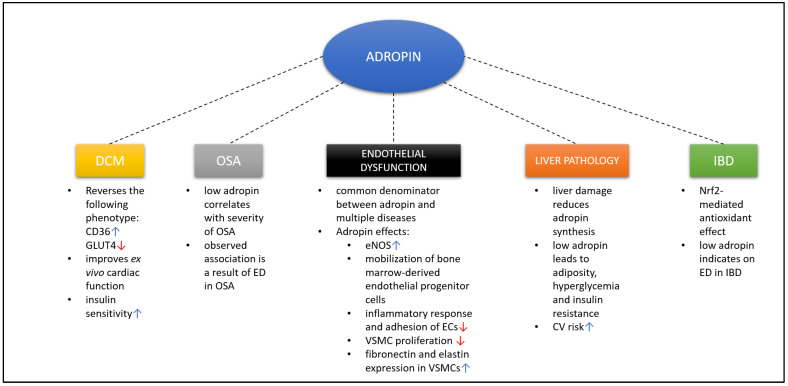
Association between adropin and different pathological states. Up arrow indicates upregulation, whereas down arrow indicates downregulation. Abbreviations: ED: endothelial dysfunction; eNOS: endothelial nitric oxide synthase; EC: endothelial cell; CV: cardiovascular; DCM: diabetic cardiomyopathy; OSA: obstructive sleep apnea; IBD: inflammatory bowel disease; VSMC: vascular smooth muscle cell; Nrf2: nuclear factor erythroid 2-related factor 2.

Overall, although adropin is implicated in a myriad of different pathologies, it seems that all of these have the same denominator—endothelial dysfunction (Figure 2).

Finally, adropin seems to be a missing link explaining the higher cardiovascular risk observed in patients with liver dysfunction [9,37]. Namely, in liver dysfunction, adropin synthesis is markedly reduced, leading to adiposity, hyperglycemia and insulin resistance [9]. Altogether, this confers a higher CV risk. In fact, a recent study suggests that patients with non-alcoholic fatty liver disease (NAFLD) display eNOS dysfunction [37]. As adropin levels in NAFLD are downregulated, it is plausible that liver pathologies result in higher CV risk via reduced adropin expression [7].

## 4. Role of Adropin in Endothelial Dysfunction

It has been well-established that the endothelium plays a central role in the maintenance of vascular homeostasis, and that impairment of endothelial function contributes to the development and progression of various pathologies, most notable with regard to the cardiovascular system [38]. eNOS bioavailability is the most important representative of endothelial function. The former is regulated by at least three distinct mechanisms: transcriptional upregulation of eNOS, posttranscriptional activation of eNOS, and reduction in ROS-mediated breakdown of nitric oxide (NO) [39]. The first study that addressed the potential endothelial protective role of adropin was conducted by Lovren et al. [16]. The authors hypothesized that adropin could represent a gatekeeper of vascular health, and thus, an integral component of cardiometabolic diseases. Their results imply that adropin affects endothelial NO synthesis by posttranscriptional stimulation (phosphorylation of Ser^1177^) of eNOS via two distinct pathways—the PI3K-Akt and ERK1/2 pathway. Nevertheless, these two pathways seem to be activated with the same upstream regulator, one of the aforementioned adropin receptors—VEGFR2. In addition, adropin may also engage several NO-independent pathways, contributing to the regulation of endothelial function, through the VEGFR2-PI3K-Akt pathway, for instance, mobilization of bone marrow-derived endothelial progenitor cells. Finally, Lovren et al. demonstrated that adropin promoted indices of endothelial function, such as proliferation, migration, capillary-like tube formation, reduced permeability and apoptosis, as well as improved angiogenic potential [16].

In the years following the above-noted study, multiple authors further explored the association between adropin and indices of ED in various clinical settings. Topuz et al. demonstrated that plasma adropin levels are reduced in type 2 diabetes mellitus (T2DM) patients with ED, as measured by brachial flow-mediated dilatation (FMD), whereas Oruc et al. demonstrated the same for patients with established metabolic syndrome using FMD as well [40,41]. Changjun et al. on the other hand associated low brain adropin levels with brain adropin pathogenesis and development of aging-associated cerebrovascular dysfunction, as Encho mRNA, adropin levels and both total and phosphorylated levels of eNOS were dramatically reduced in the aged rat brain [42]. In line with this, adropin was shown to reduce the permeability in rat brain microvascular endothelial cells under ischemic conditions and to preserve the blood–brain barrier function for intracerebral hemorrhage in mice by the Notch1 signaling pathway [43,44].

In a pivotal study by Sato et al., the authors provided the first evidence showing that adropin suppresses atherosclerosis independently of glucose and lipid metabolism, and blood pressure [45]. In fact, the study suggests that adropin exerts multiple effects in all of the three most important vascular cells participating in the pathogenesis of atherosclerosis (endothelial cells, macrophages and vascular smooth muscle cells (VSMCs)). It has already been discussed how adropin affects endothelial cells by alternating eNOS expression. Furthermore, the authors showed that adropin attenuates the inflammatory response of endothelial cells, but also of monocyte-derived macrophages, whilst attenuating monocyte-endothelial cell adhesion. Moreover, by upregulating PPAR-γ expression, adropin regulates the anti-inflammatory phenotype of monocyte differentiation. Finally, they suggested that adropin suppresses VSMC proliferation via the downregulation of the c-Src/ERK1/2 pathway, and increases fibronectin and elastin expression in VSMCs via upregulation of the PI3K-Akt pathway, thus modulating plaque stability and vascular elasticity. In the in vivo part of the above-noted study, the authors demonstrated that adropin infusion significantly reduced the development of atherosclerotic lesions in Apoe^−/−^ mice.

The clinical aspect of the role of adropin in ED has also been investigated in recent years. Multiple authors proposed that adropin might represent a novel biomarker for the presence of acute myocardial infarction (AMI), as well as predicting AMI onset in patients with coronary artery disease (CAD) [46,47]. Specifically, in a study by Wu et al., adropin was inversely and independently associated with the angiographic severity of coronary atherosclerosis, whereas adropin levels were significantly lower in patients with AMI compared with controls or patients with stable angina pectoris in a study by Yu et al. In line with this, Celik et al. reported that lower adropin levels are an independent risk factor of cardiac syndrome X, a distinctive clinical form of microvascular dysfunction [48].

Respecting the effects of adropin on NO metabolism, it was examined whether adropin has a role in blood pressure regulation. Multiple authors reported higher adropin levels in hypertensive patients compared with normotensive controls [49,50]. On the other hand, a study by Gu et al. yielded completely opposite results, with hypertensive patients having lower levels of adropin [51]. Furthermore, decreased adropin levels were found in the nocturnal hypertensive and non-dipper group of patients in a study by Bolayir et al., whereas Shelest et al. demonstrated that diminished adropin plasma levels in patients with hypertension could be a significant independent predictor of non-dipper state in hypertensive patients [52,53]. In addition, we showed significant negative correlation between adropin levels and both predialysis systolic and diastolic blood pressure in our previous study conducted on patients undergoing hemodialysis [35]. In the aforementioned study by Sato et al., adropin at doses of neither 5 nor 10 μg/kg/h showed significant effects on blood pressure in 21-week-old Apoe^−/−^ mice [45]. However, although chronic infusion of adropin at 5 μg/kg/h did not significantly impede the increase in aortic atherosclerotic lesion area and atheromatous plaque size, chronic infusion of adropin at 10 μg/kg/h significantly reduced the aortic atherosclerotic lesion area with a tendency to decrease the plaque size, as well as significantly decreasing the intra-plaque monocyte-macrophage and VSMC contents. Notwithstanding, Chen et al. postulated that adropin could have the ability to regulate blood pressure by reducing insulin resistance and adiposity, improving ED, and modulating the activity of the central nervous system [54].

As NO is the main culprit of vascular stiffness, Fujie et al. sought to determine if adropin levels are associated with vascular stiffness [55]. In the first part of the study, designed as a cross-sectional study, adropin plasma levels negatively correlated with carotid β-stiffness, an indicator of arterial stiffness, and positively with plasma nitrite/nitrate levels and cardiorespiratory fitness. On the other hand, in an intervention part of the study, adropin levels were elevated after the 8-week aerobic exercise training intervention.

## 5. Translation to Clinical Medicine and Future Perspectives

As reduced adropin plasma levels seem to be associated with various pathologies, and as they are most commonly associated with enhanced disease progression, it is merely a matter of time when adropin will be implemented in routine clinical practice. Specifically, since adropin correlates well with indices of ED, and ED being important yet poorly evaluated in clinical settings, this peptide represents a putative cardiovascular biomarker for the foreseeable future. Flow-mediated dilation is currently the most widely used method to study endothelial function. However, it is operator-dependent and can be influenced by physiological variations. Measurement of biomarkers such as adropin could overcome this issue. The biggest setback in adropin implementation is lack of understanding of the mechanisms in which it interferes with ED, thus leaving space for under recognition of confounding factors and lack of prospective large-scale studies. On the other hand, the main advantages are relatively low price, reproducibility, and according to the available data, substantial correlation with ED indices. Specifically, adropin has already shown favorable sensitivity/specificity profile for ED detection in patients with OSA.

Nevertheless, each novel biomarker has to be assessed by its appropriateness to answer elementary questions in order to judge its clinical relevance: to which group of patients should the marker be applied to, at which point in time should the marker be measured, does the biomarker provide additional information beyond existing biomarkers, and finally, whether the biomarker has yielded auspicious features in a cost-effective analysis. Hence, in order to establish adropin as biomarker of ED in different pathologies, prospective large-scale studies with appropriately defined populations are required. Notably, the signaling mechanisms and receptors by which adropin acts on different cell types also need to be fully disclosed. Better understanding of these mechanisms will alleviate implementation of adropin for both diagnostic and therapeutic purposes.

On the other hand, several authors conducted interventional studies, mostly on animals, with the intention to establish the therapeutic potential of adropin. As previously discussed, Sato et al. demonstrated that adropin exerts anti-atherosclerotic effects by suppressing monocyte–endothelial cell adhesion and smooth muscle cell proliferation. Considering the above-noted experiment, and adropin-induced upregulation of eNOS, it is plausible that adropin may be a novel target to limit a multitude of diseases in which ED is a culprit for poorer cardiovascular outcomes, exerting a vasoprotective role [16,55]. Adropin also emerged as a potential drug in the treatment of another vascular disease, but rather interestingly, not via regulation of eNOS. Notably, the role of adropin was recently explored in pulmonary hypertension, a heterogeneous group of disorders with the common feature of elevated pulmonary vascular resistance [56]. One of the most important mechanisms underlying pulmonary hypertension is the inhibition of PDH via multiple mechanisms, such as acetylation of PDH via downregulation of the SIRT3 and PDK-induced phosphorylation of PDH [57]. As PDK is upregulated in pulmonary hypertension, the search for treatment of this disease included the use of dichloroacetate, a PDK inhibitor [58]. However, in patients with SNP mutations in SIRT3, dichloroacetate was less effective [58,59]. In patients with this mutation, which is common in metabolic syndrome, reduced PDH activity is mostly a result of its acetylation of PDH [59]. Hence, bearing in mind the inhibitory effects of adropin on PDH acetylation, Bellina and Scott proposed that adropin may serve as a novel alternative to dichloroacetate for therapy of pulmonary hypertension [7].

In the setting of DCM, adropin may be beneficial as well, since adropin treatment improved ex vivo cardiac function and resulted in better glucose tolerance and reduced insulin resistance in a model of mice with diet-induced obesity [3,17]. DCM is an important target in need of novel therapeutic modalities, as despite affecting 11% of diabetics, DCM is not even recognized in routine clinical practice, rest assured treated properly [50,60]. So far, ventures of drug development for treatment of this condition were limited, most commonly due to off-target adverse effects [61]. Nevertheless, it is doubtful whether the aforementioned features of adropin would be beneficial in heart failure (HF) as a result of DCM, as studies have shown that insulin resistance actually has a beneficial role in preventing cardiac glucotoxicity in the setting of HF [62].

Finally, discussing the therapeutic implications of adropin, there is an important limitation that we need to circumvent in order to implement adropin as a therapeutic agent. Adropin being a peptide hormone, its therapeutic delivery is rather challenging, as delivery of peptide hormones is fairly limited by their pharmacokinetics and potential for proteolytic degradation. Hence, better understanding of adropin signaling pathways is mandatory, as it will pave the way for the creation of small (more resistant) molecules mimicking the effects of adropin.

## 6. Conclusions

In summary, adropin is a multifunctional highly conserved peptide implicated in various physiological and pathophysiological processes. Apart from its metabolic role, most importantly being the suppression of hepatic glucose production and improvement of insulin sensitivity, adropin seems to be an important gatekeeper of vascular health, and thus, an integral component of cardiometabolic diseases. Specifically, the vasoprotective role of adropin is achieved mainly by affecting endothelial NO synthesis. Furthermore, as plasma levels of adropin correlate with indices of ED in various pathologies and enhanced disease progression, adropin may serve as a useful biomarker in the upcoming future. Finally, the therapeutic potential of adropin in various pathologies has also been explored recently. Nevertheless, despite notable results in preliminary experiments, further well-designed studies are warranted in order to establish if adropin might be beneficial in this setting.

## Data Availability

Not applicable.
